# Hospital Meetings

**Published:** 1905-03-18

**Authors:** 


### HOSPITAL MEETINGS.

### HOSPITAL FOR DISEASES OF THE THROAT.

Mr. G. H. Pember, chairman of committee, presiding at the 41st annual general meeting of the Hospital for Diseases of the Throat, Golden Square, on the 9th inst., said the year 1904 had been an uneventful one. The grant from King Edward's Hospital Fund was £100, and from the Hospital Sunday Fund, £62 5s. 10d. Patients' contributions amounted to £4,125 5s. 9d., and there was a legacy of £50. The year closed with a balance in hand of £717 14s. 2d. The governors were greatly indebted to the energetic work of the Secretary-superintendent Mr. W. Holt, who had been instrumental in putting the finances of the hospital in the satisfactory position to which they had now attained. If anything could be done to remove the item of rates from the expenditure side of accounts, the hospital would be in a still more prosperous condition. The Council of King Edward's Hospital Fund were still urging upon the Hospital for Diseases of the Throat and the Metropolitan Ear, Nose, and Throat Hospital the desirability of amalgamation; so far, however, they had not formulated any scheme for the consideration of the various hospitals concerned; when they were in a position to do so, the matter would be carefully gone into by the Committee. With regard to economy of administration he did not fear comparison with any institution doing similar work.

The report and accounts having been passed, votes of thanks to the honorary officers, the medical and nursing staff, and the Secretary-superintendent, were unanimously passed.

### ROYAL WESTMINSTER OPHTHALMIC HOSPITAL.

Sir Charles A. Turner, K.C.I.E., presiding over the annual general meeting of Governors of the Royal Westminster Ophthalmic Hospital, said they had, in common with several other institutions, to deplore the death of his Royal Highness the Duke of Cambridge, who, as president, spared no pains to benefit the institution in every possible way. The report showed that the number of out-patients had slightly fallen off, but it was not possible to assign any special cause, though it might be due in a certain degree to the fact that a department doing similar work was now in existence at a large adjoining hospital. While other hospitals had to deplore a falling-off in subscriptions and donations, this hospital had managed to hold its own, thanks to the skill of the surgical staff and to the economy of administration. Comparison with the most prudently conducted of similar institutions was welcomed. A grant of £300 had been received from King Edward's Hospital Fund towards the reduction of the debt on the alterations, and this, with a legacy of £900 and £100 from the General Purposes Account, left the debt at the end of the year at £700. The annual cost of upkeep was some £2,400, and he was glad to be able to say that one-tenth of that amount was contributed voluntarily by the patients—*i.e.* £240. The committee were anxious again to provide when opportunity arose for patients able to pay a moderate sum towards treatment and the benefits of skilled nursing, but who were unable to afford the full cost of a private nursing home. When the hospital devoted two rooms to paying patients in the past, they were always gladly taken advantage of. With regard to the relationship existing between the hospital and the school, it must be remembered that the intention of the founder was to provide not only a hospital but a place of study for the officers of the Army and Navy. It would, therefore, be quite consistent with the original object if a portion of the funds were devoted to the school. The fees of the students were, however, sufficient to render this unnecessary, the only expense incurred being the small cost of lighting the lecture hall. It was largely owing to the liberality of the surgical staff that this happy state of things was possible, and they had, by voluntarily fitting up the surgery during the recent alterations, given a striking instance of their generosity.

The report and accounts having been passed, Miss M. M. Evans deplored the growing competition of outside bodies in doing special work such as that carried on by the hospital, cases formerly sent to this hospital by the Poor-law Guardians being now dealt with largely by the Metropolitan Asylums Board. Other speakers having borne testimony to the excellent quality of the work of the surgical staff, to whom with other officers hearty votes of thanks were passed, Prebendary Shelford asked whether it would not be in accordance with custom to acknowledge the services, as hon. Chaplain, of the senior Curate of St. Martin's-in-the-Fields, who visited the hospital regularly throughout the year, but whose name did not appear in the list of officers. The chairman explained that the name of the Chaplain was omitted by the wish of the late Prebendary Kitto, and promised that it should be restored to the report. The meeting closed with a warmly appreciative vote of thanks to the chairman for his devotion to the interests of the institution.

### NATIONAL HOSPITAL FOR DISEASES OF THE HEART.

THE EARL OF VERULAM, presiding at the 48th annual meeting of the National Hospital, Soho Square, referred to a recent visit from King Edward's Hospital Fund, the result of which was a grant of £15 to complete the purchase of the *x*-ray apparatus. This had been installed, and was proving of great assistance to the medical staff. There had been an increase in the grants from both the Sunday and Saturday Funds, these being in 1904, £131 5s. 10d. and £92 8s. respectively. As shown in the balance sheet, it had been necessary to transfer £200 from the interest on the Building Fund, in order to meet current expenses and avoid a deficit. This was, however, unsatisfactory, and he felt it to be very desirable that the annual income should be so augmented as to enable current expenses to be met by current receipts. By the death of Dr. Norman B. Elliot they had lost a courteous and sympathetic member of the medical staff, whose connection with the hospital extended over several years. The thanks of the governors were due to the medical and nursing staff, and to the honorary officers, as well as to many donors of presents for the use of the patients, including two children's cots. Dr. Chapman said the gift of some bed-tables would be greatly appreciated; he hoped also that it would be remembered that the new *x*-ray apparatus was expensive in up-keep; donations for that purpose were very necessary. The want of accommodation for out-patients continued to be greatly felt by the medical staff, who were obliged to refuse applicants on that account.

The report and accounts having been adopted, the usual votes of thanks were passed, and with a warm appreciation of the services rendered by the chairman in the interests of the hospital, the meeting terminated.

### WEST END HOSPITAL.

THE annual general meeting of the West End Hospital for Diseases of the Nervous System, Paralysis, and Epilepsy, was held on the 3rd inst.

The Chairman, Major Strachan-Davidson, read the report and drew attention to the fact that the subscriptions were well maintained, but that donations had fallen off.

This year £125 have been received from King Edward's Fund to be spent towards the erection of a balcony for the children—the only means of giving the little ones fresh air in the closely built district in which the hospital stands. The Hospital Sunday Fund granted £258, and the Hospital Saturday Fund contributed £158. An anonymous contributor sent £100 through Dr. Outterson Wood. In spite however of these substantial helps the Hospital's banking account is £300 on the wrong side.

Heavy expenses have been incurred in building and in improved precautions against fire. The hospital has no endowment beyond £100 in Consols, and the committee hope that the public will contribute generously towards wiping off the debt. The isolation ward has been costly to build, but nearly all the debt is paid, and it is hoped that, ere long, means may be found to admit adult patients, who at present, however urgent their need, can only be treated in the out-patient department. A vote of thanks to the medical staff was passed, and, in replying to it, Dr. Outterson Wood gave a generous tribute to the zealous work and devotion to the hospital of the treasurer, Mr. H. C. Willock. He also spoke of the good work done by the late matron, Mrs. H. Brown, who left the hospital in July last, and congratulated the committee of management upon the highly satisfactory appointment of Miss Thorpe to the vacant post.

